# Precoder Design for Network Massive MIMO Optical Wireless Communications

**DOI:** 10.3390/s24165188

**Published:** 2024-08-11

**Authors:** Zakir Ali, Chen Sun, Qasim Jan, Muhammad Furqan, Xiqi Gao

**Affiliations:** 1 National Mobile Communications Research Laboratory, Southeast University, Nanjing 210096, China; sunchen@seu.edu.cn (C.S.);; 2 Department of Computer Sciences, Bahria University Lahore Campus, Lahore 54000, Pakistan; 3 Purple Mountain Laboratories, Nanjing 211111, China; 4 Department of Computer Science, National University of Computer and Emerging Sciences Peshawar Campus, Peshawar 25100, Pakistan; qasim.jan@nu.edu.pk; 5 Department of Software Engineering, Capital University of Science and Technology (CUST), Islamabad 44000, Pakistan; muhammad.furqan@cust.edu.pk

**Keywords:** network massive MIMO, optimization, OWC, precoder design, sum rate

## Abstract

Precoding is a technique employed to enhance transmission rates in various communication systems, including massive multiple-input multiple-output (MIMO) and optical wireless communication (OWC). In this study, we focus on network massive MIMO OWC (NM-MIMO-OWC) systems and investigate the precoder design to enhance the sum rate and improve the system performance. We present the network’s massive MIMO OWC framework. By utilizing this framework, we are able to calculate the achievable sum rate. Subsequently, we consider the precoding design for maximizing the sum rate while adhering to the total power constraint. To solve this optimization problem, we provide a necessary condition of the optimal solution based on the Karush–Kuhn–Tucker (KKT) conditions, and propose a low-complexity algorithm to further enhance the efficiency of the proposed precoding technique. The numerical results demonstrate that the proposed precoder design significantly improves the transmission rate and effectively maximizes the sum rate.

## 1. Introduction

An explosive evolution occurs in the usage of applications using IoT (Internet of things) devices in different fields like engineering, medicine, agriculture, industry, transportation, etc., leading to the widespread requirement of wireless communications [1]. By the end of 2023, nearly two-thirds of the global population had access to the internet, and the number of connected devices exceeded three times the world’s population [2]. It has been projected that the use of linked network devices all over the world will reach up to 75 billion by 2025, a threefold increase from 2019 [3]. Wireless communication is developing rapidly to achieve boosted mobile broadband, ultra-low latency communication, and giant connectivity to meet the design goal of fifth-generation (5G) and beyond 5G (B5G) networks [4]. Wireless traffic is increasing at an exponential rate, and applications in the next-generation network, such as holographic communications, robotics and autonomous driving, mobile edge computing (MEC), augmented reality (AR), virtual reality (VR), chip-to-chip communication, unmanned aerial vehicles (UAV), and so on, necessitate a very high data rate. 5G, which is based on the traditional radio frequency (RF) system, attempts to meet the requirements of the aforementioned applications but it is incapable of providing elevated energy efficiency and maximum crowded connection support for these communication services due to its limited spectrum resources [5,6].

The rapid increase in wireless traffic requires an additional spectrum to provide network connectivity to everything on the earth (global coverage). Optical wireless communication (OWC) is considered as a supplementing technology for the sixth generation (6G), which supports a higher data rate, robust security, and is more energy efficient [7,8,9]. To support multi-user communications, multi-user multiple-input multiple-output (MU-MIMO), or even massive MIMO, is applied in OWC. By combining OWC with MU-MIMO, we can achieve enhanced network capacity and throughput, improved quality of service, and cost-effective deployment advantages in future communication systems [10,11,12].

In OWC systems, the coverage and communication capability of one base station (BS) is usually limited. Multiple BSs simultaneously communicate with multiple user terminals (UTs), forming a network massive MIMO OWC (NM-MIMO-OWC) system, which further increases the transmission rate of each UT and ensures that all UTs receive uninterrupted service [13]. In the NM-MIMO-OWC system, multiple transmitters independently send the signal to all UTs simultaneously, resulting in multi-user interference (MUI) and multi-cell interference (MCI). A better solution to reduce MUI, as well as MCI, is to jointly design the transmitted signals. In contrast, the nature of the optical signal is different from the RF signal. Some modification is needed to use the precoding techniques of RF in the OWC [14,15]. Few precoding approaches are considered in MU-MIMO OWC, massive MIMO OWC, and network MIMO systems.

### 1.1. Literature Review

In order to address the challenge of MUI and MCI, some studies have explored precoding techniques. The following works studied MU-MIMO OWC systems. The work in [16], introduced a new MU-MISO visible light communication (VLC) system intended to reduce the MUI, while providing a consistent beam. They first formed a suitable transceiver under a specific per-laser emitting diode (LED) optical power constraint and proposed a condensed transceiver based on zero-forcing transmitter precoding. A precoding technique based on a block diagonalization algorithm for the MU-MIMO system in indoor VLC is presented in [17]. The study in [18], evaluated the performance of MU-MIMO precoders for indoor VLC systems to reduce the transmitted optical power under per LED and imperfect CSI constraints. The transmitter for VLC broadcasting signal to multiple users, and the the synchronization possibility of each sender, i.e., LED, are studied and improved linear precoding filters are presented using the minimum mean square error (MMSE) technique while considering the per LED optical power constraint. It is stated that using a low-rate feedback channel, the channel state information (CSI) of the transmitter is obtained, and a robust precoder design is proposed [19].

Building upon the above works on MU-MIMO, we explore some recent studies, including fiber-enabled OWC and beam domain MIMO-OFDM OWC systems. A novel massive MIMO transmission in a beam for OWC has been studied and presented with a linear precoding design for sum-rate maximization. With the exponential increase in the number of transmitters, their proposed technique of beam division multiple access (BDMA) transmission gives better results under total and per transmitter constraints [20]. The researchers in [21], proposed an asymptotic optimum algorithm for a large number of fiber ports in a fiber-enabled optical wireless communication (FE-OWC) system. MMSE-based precoding to improve the sum rate in the massive MIMO optical wireless communication system was explored [22]. A beam domain MIMO-OFDM and an iterative algorithm based on the sequential parametric convex approximation (SPCA) method in MIMO OWC with peak-to-average power ratio (PAPR) have been analyzed, and that showed the enhanced transmission rate and minimized PAPR [23].

We discuss various precoders for massive MIMO systems aimed at reducing MUI and MCI improving sum rate, and optimizing performance under different constraints. The authors in [24] studied weighted minimum mean square error (WMMSE) robust precoding for downlink with imperfect CSI and deep learning design for massive MIMO communications. The study delves into the design of a linear precoder for maximizing the weighted sum rate (WSR) in a massive MIMO downlink single-cell system with multiple users and introduces a unified matrix manifold optimization framework [25]. A low-complexity signal detection algorithm based on the successive overrelaxation (SOR) method was considered deprived of complex matrix inversion to obtain the near best performance to the MMSE algorithm and examined the performance of the SOR technique on different features [26].

Expanding on the previously discussed techniques, the following works investigate various precoding approaches specifically for network massive MIMO systems. These techniques include bisection search and block diagonalization. A simple bisection-based algorithm is proposed in the multicell cooperative transmission environment, called network-MIMO and an effective precoding matrix is investigated by using the WMMSE criterion to obtain sum rate maximization in the simultaneous wireless information and power transfer (SWIPT) network MIMO [27]. An asymptotic expression of rate with zero forcing criteria in multicell network MIMO with MUI is considered and proposed as an algorithm that can calculate the output by proportional fairness (PF) method and is showed by the expansion of an appropriate concave and module-wise growing the effective function of the network [28]. To intensify the sum rate and restrict inter-cluster coordination to decrease interference for cluster edge users, the authors proposed a base transceiver station synchronized method divided into clusters and designed a precoder based on multi-cell block diagonalization for large MIMO, i.e., network MIMO [29]. Furthermore, BDMA achieves high throughput in a networked optical massive MIMO system having several BSs with transmit lenses and an optical transmitter array simultaneously serving various UTs [30]. The previously mentioned works dealt with sum-rate maximization for network massive MIMO systems, whereas, to the best of our knowledge, none have considered network OWC systems.

### 1.2. Contributions

This paper presents a novel precoder design for network massive MIMO OWC, where, multiple BSs are equipped with massive optical transmitters, enabling simultaneous communication with many UTs. Our study involves calculating the achievable sum rate, determining the necessary conditions for optimal precoder design through Karush–Kuhn–Tucker (KKT) conditions, and proposing an iterative algorithm. The main contributions of this paper are summarized as:

- We develop a network massive MIMO OWC systems. We first provide the network’s massive MIMO system model. Multiple optical transmitters radiate light in diverse directions by utilizing a transmit lens at every BS; meanwhile, the light from diverse directions is refracted and accepted by various photodetectors at the UT side with the receiving lens. Every BS transmits autonomous signals to various UTs, and every UT isolate signals received from different BSs. It shows a significant prospect of a network of massive MIMO OWC systems.
- We derive the necessary conditions of the optimal precoding to maximize the sum rate. We calculate the achievable rate of the network’s massive MIMO communication OWC system. We formulate a sum rate maximization problem with total power constraint. By employing the KKT conditions, we provide the optimality conditions for sum-rate maximization.
- We propose an iterative precoder design for sum-rate maximization satisfying the total power constraint. Based on the optimality conditions, we propose an iterative algorithm that finds the optimal Lagrangian multiplier and achieves the optimal precoder design. The numerical results validate the better performance of the proposed technique.

### 1.3. Organization of Paper

The rest of the paper is organized as follows: In Section 2, we provide a detailed overview of the system model, including system configuration, analysis of optical transmitters and receivers, and the signal model. Problem formulation, the optimal structure of precoder for transmitter and receiver, and the KKT conditions for the sum rate maximization are investigated in Section 3. Section 4 describes the proposed Iterative Precoder design under power constraint. The numerical results are presented in Section 5. Finally, Section 6 concludes the paper.

### 1.4. Notations

In this manuscript, we employ the following notations:

- $R^{N\times M}$ denotes the N×M dimensional real matrix space.
- The upper bold letters denote matrices, lower bold letter represents coloumn vectors and I is the identity matrix.
- Superscript ${\left(.\right)}^{T}$${\left(.\right)}^{H}$ denotes the transpose and conjugate transpose of a matrix.
- $\mathrm{tr}\left(.\right)$, $\det\left(.\right)$, error(.) and ℜ(.) express the trace of a matrix, determinant, expected value, and real part, respectively.
- ${\left[.\right]}_{i}$ indicates the *i*-th element of a vector, and ${\left[A\right]}_{i,j}$ represents the (i,j)-th element of matrix A.

## 2. System Model

In this section, we provide an introduction to the system configuration, an analysis of the optical transmitter/receiver and the signal model, which effectively illustrates the behavior of the signal transmission, and the last part discusses the achievable rate.

### 2.1. System Configuration

**Figure 1:**
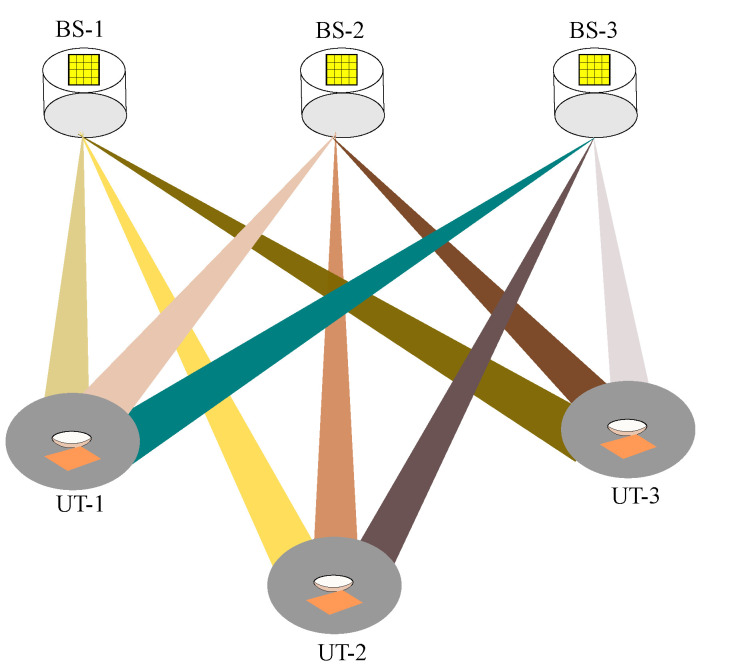
NM-MIMO-OWC downlink system.

Consider a network massive MIMO OWC system, where *B* BSs simultaneously communicate with *U* UTs as presented in Figure 1. Each BS is equipped with *M* optical transmitters, and each UT has *N* optical receivers. Afterward, we interpret the properties of the optical transmitter and receiver and establish the system model of the network massive MIMO OWC.

### 2.2. Analysis of Optical Transmitter and Receiver

In this sub-section, we describe the optical transmitter and receiver components in our proposed network massive MIMO OWC system, focusing on the behavior of the transmitted signal from the transmitter to the receiver. In network massive MIMO OWC systems, the incorporation of multiple transmitters and receivers enables the achievement of high data throughput and reliable communication connections. This approach utilizes a spatial multiplexing technique to simultaneously transmit and receive multiple data streams, thereby improving the system’s overall capacity and spectral efficiency. Each optical source is modulated independently with its corresponding data stream, enabling the simultaneous transmission of multiple data streams. The transmitted beams from any optical transmitter of a BS pass through a transmit lens to control the divergence and directionality of the transmitted optical beams. Intensity modulation and direct detection (IM/DD) techniques are utilized to encode data into optical signals. In practical applications, the beam emitted by a single optical transmitter tends to be concentrated within a narrow-angle. When the emitted angle is larger, the beam intensity decreases significantly. Our consideration is directed towards the beam within a specific angle. This particular beam, generated by a transmitting optical antenna with a specific emitted angle, allows us to accurately describe the optical intensity of the refracted light in relation to the relative refractive angle [11,12].

The optical receiver typically consists of an array of photodetectors, such as photodiodes (PDs) or avalanche photodiodes (APDs). Each PD is responsible for receiving one of the transmitted optical beams, allowing for parallel data reception. An optical concentrator that comes before a detector and preamplifier is used to collect and direct the incoming optical signals onto the PD array. The photodiode array receives the beams emitted by the optical transmitter, causing them to refract and convert them into an electrical signal. It is rare that sometimes PD has a specific constraint, allowing it to receive only a fraction of the optical beam transmitted by a single transmitter [30,31].

As the BS is equipped with multiple optical transmitters, the beams are refracted to disparate sides, producing optical signals in different directions, effectively covering the entire communication zone. Optical receivers are used at every UT so that optical signals (light) from different BSs are refracted to various photodetectors and set up the signal model for the network massive MIMO OWC system.

### 2.3. Signal Model

In network massive MIMO OWC system, different BSs transmit independent signals to different UTs. The transmitted signal is the data stream passing through a precoding matrix. The downlink received signal $r_{i}\in R^{N\times 1}$ at the *i*-th UT can be expressed as follows:

(1) $$r_{i}=\sum_{\ell }H_{i,\ell }P_{i,\ell }x_{i,\ell }+\sum_{\ell }\sum_{k\neq i}H_{i,\ell }P_{k,\ell }x_{k,\ell }+n_{i},$$

where $H_{i,\ell }\in R^{N\times M}$ is the channel matrix representing the communication link between the *ℓ*-th BS and the *i*-th UT, $P_{i,\ell }$ is the transmit precoding matrix, while $x_{i,\ell }\in R^{s\times 1}$ denotes the transmitted signal of UT *i*, where *s* is the number of data streams and $n_{i}$ expresses the additive white Gaussian noise elements with independently and identically distributed (i.i.d.) variables with zero mean and variance $\sigma^{2}$.

The opening value on the right-hand side of (1), i.e., $\sum_{\ell }H_{i,\ell }P_{i,\ell }x_{i,\ell }$, resembles the expected signal, while $\sum_{\ell }\sum_{k\neq i}H_{i,\ell }P_{k,\ell }x_{k,\ell }+n_{i}$ represents inter-user interference and additive white Gaussian noise. We consider that CSI is available to BSs and all UTs. The *i*-th UT treats the aggregate interference-plus-noise as the equivalent Gaussian noise with the covariance as:

(2) $$\begin{array}{cccccccccc} R_{i}=\sum_{\ell }\sum_{k\neq i}H_{i,\ell }P_{k,\ell }P_{k,\ell }^{T}H_{i,\ell }^{T}+\sigma_{n}^{2}I. & \; & & \; & & \; & & \; & & \; \end{array}$$

We calculate the achievable rate for UT as:

(3) $$\begin{array}{cc} \; & R_{i}=\frac{1}{2}\log\det\left(\sum_{\ell }\sum_{k}H_{i,\ell }P_{k,\ell }P_{k,\ell }^{T}H_{i,\ell }^{T}+\sigma_{n}^{2}I\right)\\ & -\frac{1}{2}\log\det\left(\sum_{\ell }\sum_{k\neq i}H_{i,\ell }P_{k,\ell }P_{k,\ell }^{T}H_{i,\ell }^{T}+\sigma_{n}^{2}I\right). \end{array}$$

**Remark 1.**  *The above equation represents the achievable rate for a UT in an NM-MIMO-OWC system, where multiple users communicate with multiple BSs simultaneously. The summations over ℓ and k account for the contributions from all transmit antennas and UTs, respectively. The main difference between RF and OWC systems is that OWC employs IM/DD and considers only real-valued signals, which results in a factor of 1/2. From (3), it is apparent that the achievable rate of UT i depends on the precoding matrices of all the BSs. Next, we consider the precoding matrix design.*

## 3. Necessary Conditions for Optimal Precoding

In this section, we begin by presenting the problem formulation for maximizing the sum rate in the design of network massive MIMO OWC, while considering the power constraint of each BS. Then, we derive the necessary conditions for the optimal precoding design based on KKT conditions to solve a non-convex optimization problem.

### 3.1. Problem Formulation

In this subsection, we consider the problem of maximizing the sum rate of a network massive MIMO OWC system, while adhering to a single BS power constraint. One way to tackle utility maximization problems is through the formulation of a sum rate maximization problem, which is expressed as:

(4) $$\begin{array}{ccc} \; & \max_{P_{j,m}}\sum_{i=1}^{U}R_{i},\\ \; & \mathrm{subject}\;\mathrm{to}:\sum_{i=1}^{U}\mathrm{tr}\left(P_{i,\ell }P_{i,\ell }^{T}\right)\leq P. & \; \end{array}$$

In this particular scenario, our primary goal is to maximize the sum rate $R_{i}$ by determining the optimal values of the precoding matrix $P_{j,m}$. We aim to determine the solution to the sum rate maximization problem, subject to the condition that the total power of one BS allocated to all users should not exceed power constraint *P*.

We rewrite the sum rate maximization problem in (4) by substituting the value of $R_{i}$ from (3), while keeping the same power constraint. The resulting equation is expressed as:

(5) $$\begin{array}{c} \max_{P_{j,m}}\sum_{i=1}^{U}\frac{1}{2}\{\log\det\left(\sum_{\ell }\sum_{k}H_{i,\ell }P_{k,\ell }P_{k,\ell }^{T}H_{i,\ell }^{T}+\sigma_{n}^{2}I\right)\\ -\frac{1}{2}\log\det\left(\sum_{\ell }\sum_{k\neq i}H_{i,\ell }P_{k,\ell }P_{k,\ell }^{T}H_{i,\ell }^{T}+\sigma_{n}^{2}I\right)\}, \end{array}$$

$$\begin{array}{c} \mathrm{subject}\;\mathrm{to}:\sum_{i=1}^{U}\mathrm{tr}\left(P_{i,\ell }P_{i,\ell }^{T}\right)\leq P. \end{array}$$

Here, the precoding matrix $P_{j,m}$ is selected to enhance the achievable data rate, $R_{i}$ for all users, subject to the original constraint in (4). The presence of quadratic terms of $P_{j,m}$ inside the log determinant operation, combined with the minus sign in front of the second logarithmic determinant term, changes the sign of the quadratic term, rendering the overall objective function non-convex, making it challenging to find the global optimum.

**Remark 2.**  *Different power control strategies in NM-MIMO-OWC systems significantly impact system performance compared to the total power constraint (TPC). The Per-Antenna Power Constraint (PAPC) and the Per-User Power Constraint (PUPC) offer more practical and realistic approaches. However, these approaches involve certain trade-offs that need careful consideration in the design and implementation of NM-MIMO-OWC system. PAPC, which limits the power of each antenna, is more aligned with the hardware limitations of individual power amplifiers in practical systems. This constraint leads to reduced flexibility in power allocation compared to TPC, potentially resulting in lower overall system capacity. However, PAPC ensures a more balanced power distribution across antennas, which is beneficial for energy efficiency and hardware longevity. On the other hand, PUPC focuses on limiting power allocated to each user, which is particularly useful in multi-user scenarios for ensuring fairness and quality of service. While PUPC may not achieve the maximum sum-rate capacity that TPC could theoretically provide, it offers better control over individual user performance. In our proposed NM-MIMO-OWC system, where the number of antennas is significantly large, the impact of these constraints becomes more pronounced. The high spatial diversity partially compensate for the limitations imposed by PAPC or PUPC, but the system may still experience some performance degradation compared to the TPC. Consequently, the choice between these power control strategies in the NM-MIMO-OWC system involves a trade-off between theoretical performance, practical implementation considerations, and specific system requirements.*

### 3.2. Optimal Structure of Precoder

The objective function and constraint in the problem (5) is non-convex and cannot be solved directly using convex optimization techniques. By introducing Lagrangian multipliers, the problem can be solved, and the Lagrangian is expressed as:

(6) $$L\left(P_{j,m},\lambda \right)=\sum_{i=1}^{U}R_{i}-\sum_{\ell }\lambda_{\ell }\left(\sum_{i=1}^{U}\mathrm{tr}\left(P_{i,\ell }P_{i,\ell }^{T}\right)-P\right),$$

where $\lambda_{\ell }$ is the Lagrangian multiplier. Now, if we substitute the value of (3) into (6), we obtain:

(7) $$\begin{array}{cc} \; & L(P_{j,m},\lambda )=\\ & \sum_{i=1}^{U}\frac{1}{2}\{\log\det\left(\sum_{\ell }\sum_{k}H_{i,\ell }P_{k,\ell }P_{k,\ell }^{T}H_{i,\ell }^{T}+\sigma_{n}^{2}I\right)\\ & -\frac{1}{2}\log\det\left(\sum_{\ell }\sum_{k\neq i}H_{i,\ell }P_{k,\ell }P_{k,\ell }^{T}H_{i,\ell }^{T}+\sigma_{n}^{2}I\right)\}\\ & -\sum_{\ell }\lambda_{\ell }\left(\sum_{i=1}^{U}\mathrm{tr}\left(P_{i,\ell }P_{i,\ell }^{T}\right)-P\right). \end{array}$$

Utilizing the matrix formula from [32], we proceed to compute the partial derivative of L with respect to $P_{j,m}$ as:

(8) $$\begin{array}{c} \frac{\partial L}{\partial P_{j,m}}=\sum_{i}A_{i,m}P_{j,m}-\sum_{i\neq j}B_{i,m}P_{j,m}+\lambda_{\ell }P_{j,m}, \end{array}$$

where $A_{i,m}$ and $B_{i,m}$ are given in (9) and (10), respectively:

(9) $$A_{i,m}=H_{i,m}^{T}{\left(\sum_{\ell }\sum_{k}H_{i,\ell }P_{k,\ell }P_{k,\ell }^{T}H_{i,\ell }^{T}+\sigma_{n}^{2}I\right)}^{-1}H_{i,m},$$

(10) $$B_{i,m}=H_{i,m}^{T}{\left(\sum_{\ell }\sum_{k\neq i}H_{i,\ell }P_{k,\ell }P_{k,\ell }^{T}H_{i,\ell }^{T}+\sigma_{n}^{2}I\right)}^{-1}H_{i,m}.$$

Based on the aforementioned derivation and KKT conditions, the optimal precoding satisfies the following conditions.

**Theorem 1.**  *The optimal precoding matrix satisfies:*

(11) $$\begin{array}{c} \sum_{i}A_{i,m}P_{j,m}-\sum_{i\neq j}B_{i,m}P_{j,m}-\lambda_{\ell }P_{j,m}=0, \end{array}$$

*which can be rewritten as:*

(12) $$\begin{array}{c} P_{j,m}={\left(\sum_{i}B_{i,m}-\sum_{i}A_{i,m}+\lambda_{\ell }I\right)}^{-1}B_{j,m}P_{j,m}. \end{array}$$

**Proof.**  The proof is provided in Appendix A.    □

**Remark 3.**  *Theorem 1 provides the necessary conditions for the optimal precoding matrix* $P_{j,m}$ *, as it does not have a closed-form solution. The* $\lambda_{\ell }$ *plays a crucial role in determining the optimal value, and it is calculated for each BS to obtain the optimal result. Hence, there is no closed-form expression for* $P_{j,m}$ *; therefore, we calculate it iteratively according to (12).*

## 4. Iterative Precoder Design Algorithm

This section presents an iterative precoder design algorithm. It explains the various steps of the proposed algorithm and concludes with a discussion of the computational complexity of the proposed technique.

Algorithm 1 outlines an iterative approach for computing the optimal precoding matrices in the proposed NM-MIMO-OWC system under a total power constraint. It employs a bisection search to find the optimal value of a parameter $\lambda_{\ell }$ used in the iterative precoder design. The steps are explained as:

- All the channel matrices, $H_{i,\ell }$, the noise variance $\sigma^{2}$, and total power constraint *P* are given as input. The precoding matrices which fulfilled the power constraint are then initiated.
- The algorithm starts with an interval by setting lower and upper bounds, i.e., $\lambda_{\min}$ and $\lambda_{\max}$ for the bisection search and starts searching through each BS from 1 to L. For each BS, it performs a bisection search to find the optimal value of $\lambda_{\ell }$ by dividing the search interval in half, i.e., $\frac{1}{2}\left(\lambda_{\min}+\lambda_{\max}\right)$. The algorithm computes the midpoint $\lambda_{\ell }$ of the current search interval $\lambda_{\min},\lambda_{\max}$. Using the current value of $\lambda_{\ell }$, it computes the optimal precoding matrix $P_{j,m}$ using Equation (12).
- The algorithm checks whether the current computed power *p* is greater than the total power *P*. If it is, the algorithm updates the lower bound $\lambda_{\min}$ to the current value of $\lambda_{\ell }$. Otherwise, it updates the upper bound $\lambda_{\max}$ to the current value of $\lambda_{\ell }$. The algorithm repeats steps 5–7 until the difference between $\lambda_{\max}$ and $\lambda_{\min}$ is less than the convergence limit ϵ. The algorithm repeats steps 3–11 for all BSs. The algorithm checks for convergence or reaches a predefined value. If the algorithm converges, it terminates. Otherwise, it repeats the entire process until convergence or the maximum number of iterations is reached. All the steps are summarized in Algorithm 1.

**Algorithm 1** Iterative Precoder Design for Network Massive MIMO OWC System.     **Input:** The channel matrix H, the noise variance $\sigma^{2}$ and total power constrant *P*.     **Output:** The precoding matrix $P_{j,m}$.1:Initialize the precoding matrix satisfying the power constraint $\sum_{i=1}^{U}\mathrm{tr}\left(P_{i,\ell }P_{i,\ell }^{T}\right)\leq P$.2:**for**  l=1,..., L **do**3:    Initialize variables for bisection search: $\lambda_{\min}$, $\lambda_{\max}$.4:    **while** $\lambda_{\max}-\lambda_{\min}>\epsilon$ **do**5:        Compute $\lambda_{\ell }=\frac{1}{2}\left(\lambda_{\min}+\lambda_{\max}\right)$.6:        Compute the optimal precoding matrix $P_{j,m}$ by using (12).7:        Calculate total power *p*.8:        **if** p>P **then**9:           $\lambda_{\min}=\lambda_{\ell }$10:        **else**11:           $\lambda_{\max}=\lambda_{\ell }$12:        **end if**13:    **end while**14:    **until**: convergence or predefined value.15:**end for**

The algorithm iterates between optimizing the precoders for the current $\lambda_{\ell }$ and updating $\lambda_{\ell }$ via bisection search to satisfy the total power constraint, until convergence.

### Analysis of Computational Complexity

The computational complexity of the proposed precoder design is $O(BUM^{3})$ per iteration, where *B* is the number of BSs, *U* denotes the number of UTs and *M* represents the number of transmit optical antennas. Considering the number of receiver optical antennas is much less than that of transmit optical antennas, which grows cubically with the number of optical antennas. The per-iteration nature of the complexity implies that the total computational burden is influenced by the convergence rate of the algorithm. The iterative percoder design in (12) requires a matrix inversion operation, whose dimension is *M*. Thus, the complexity of one iteration is $M^{3}$. Each BS needs to calculate the precoder for *B* BSs and *U* UTs; therefore, the complexity of the proposed iterative precoder is $O(BUM^{3})$.

The complexity increases linearly with the number of BSs and UTs. The linear relationship shows that the computational requirements of the algorithm grow in proportion to the size of the network and the density of users. This scalability is useful for deployment in various large-scale scenarios. The computational complexity of the algorithm is determined by the number of iterations required to converge. The simulation results show that the proposed scheme converges quickly, which indicates the effectiveness and efficiency of the proposed precoder design in reaching a solution within a short time frame. This rapid convergence demonstrates the robustness of the proposed algorithm in various scenarios. The cubic term $M^{3}$ is of particular significance, as it dominates the complexity scaling. The proposed precoder design is anticipated to offer better interference management, enhanced spectral utilization, and increased system capacity in comparison to less computationally precoding techniques. The proposed precoder design has the capability to address the challenge of multi-user interference in a large-scale system.

## 5. Numerical Results

In this section, we present numerical results to validate the effectiveness of our proposed iterative-based precoder design in a network massive MIMO OWC system. We consider a network having three BSs; each BS is equipped with 128 optical antenna transmitters arranged in an array. These BSs communicate with 10 UTs, which are positioned differently within a room measuring 4 m × 4 m × 2 m. Simulation parameters are summarized in Table 1.

**Figure 2:**
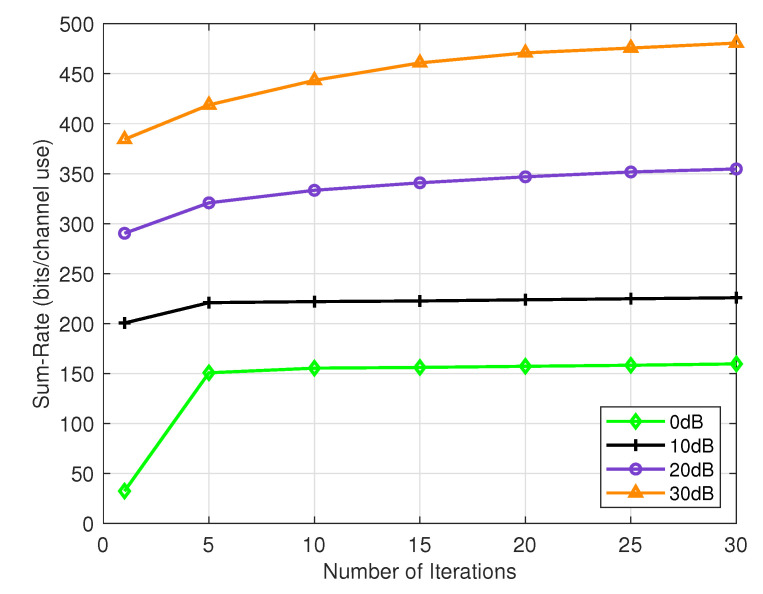
Convergence behavior of the proposed algorithm.

Figure 2 illustrates the convergence behavior of our proposed algorithm across various signal-to-noise ratios (SNRs) ranging from 0 dB to 30 dB. The results demonstrate the algorithm’s ability to converge rapidly, even with low total power. Notably, at 0 dB SNR, the algorithm achieves convergence with a higher sum rate in a minimal number of iterations. Similarly, at 10 dB and 20 dB SNR, the algorithm’s performance remains consistent, requiring fewer iterations while delivering a superior sum rate performance. However, even at 30 dB SNR, the proposed algorithm still requires fewer iterations to achieve better performance.

We compare the sum rate of our proposed precoder with two widely used precoders in wireless communications: maximum ratio transmission (MRT) and regularized zero-forcing (RZF) precoders. The precoding vectors for MRT and RZF are defined as follows [23,30]:

(13) $$\begin{array}{c} w_{k,\ell }^{\mathrm{MRT}}=\sqrt{\beta_{\ell }^{\mathrm{MRT}}}h_{k,\ell }, \end{array}$$

(14) $$\begin{array}{c} w_{k,\ell }^{\mathrm{RZF}}=\sqrt{\beta_{\ell }^{\mathrm{RZF}}}{\left(H_{\ell }H_{\ell }^{T}+\alpha^{\mathrm{RZF}}I\right)}^{-1}h_{k,\ell }. \end{array}$$

where $\beta_{\ell }^{\mathrm{MRT}}$ and $\beta_{\ell }^{\mathrm{RZF}}$ is the power factor to satisfy the power constraint and $\alpha^{\mathrm{RZF}}$ is the regularized factor.

**Figure 3:**
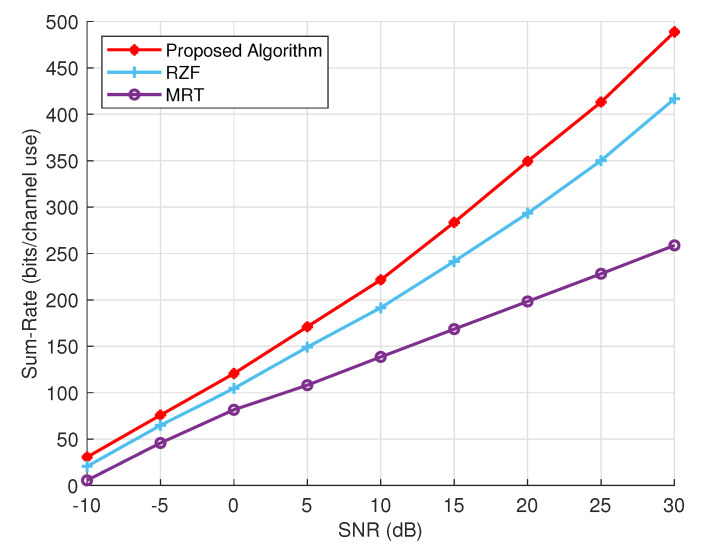
The sum rate performance of the proposed algorithm versus RZF and MRT at different SNRs.

We present a comparison of the achievable sum rates of the conventional and the proposed precoding schemes in Figure 3. The performance of these techniques varies from the outset, particularly at low SNR, showing a noticeable difference. This difference becomes more pronounced as the SNR increases. At higher SNR levels, the performance of MRT declines compared to RZF. Overall, RZF demonstrates a superior sum rate compared to MRT, especially at high SNR levels. In contrast, the proposed precoder outperforms both precoders in both low and high SNR regions. Our proposed precoder notably achieves a significantly higher sum rate in the high SNR region, with a consistent increase observed ranging from 5 dB to 20 dB SNR. At 25 dB and 30 dB SNR, the sum rate experiences a significant boost in performance.

**Figure 4:**
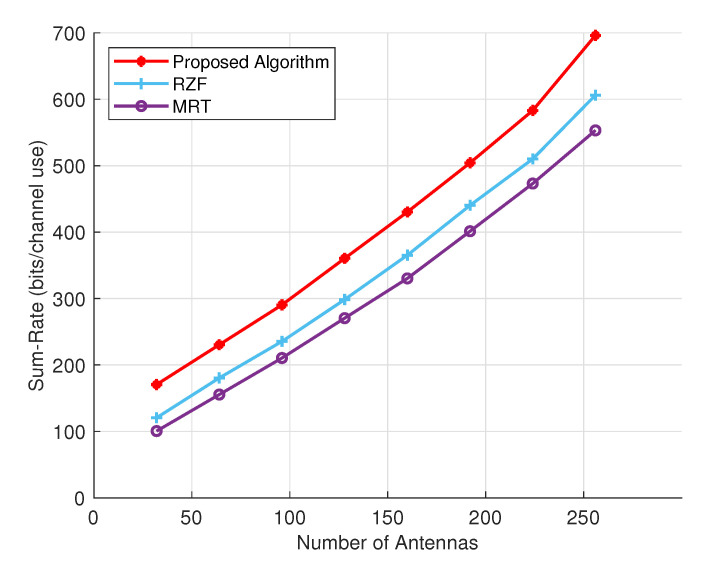
Perceiving the relationship: Comparing transmitting optical antennas to the sum rate.

Figure 4 shows the achievable sum rate performance, measured in bits per channel use, for varying numbers of transmit antennas. The proposed method, starting with 32 antennas, achieves approximately 170 bits per second. In comparison, the RZF method yields around 120 bits per second, and the MRT method reaches about 100 bits per second. With an increased number of antennas, the BS gains improved spatial resources, allowing for communication with multiple UTs. As the number of transmit antennas grows, the proposed technique demonstrates superior performance in terms of sum rate when compared to other precoding methods. Even with 256 transmit antennas, the proposed precoding method continues to exhibit significantly better performance in sum rate.

**Figure 5:**
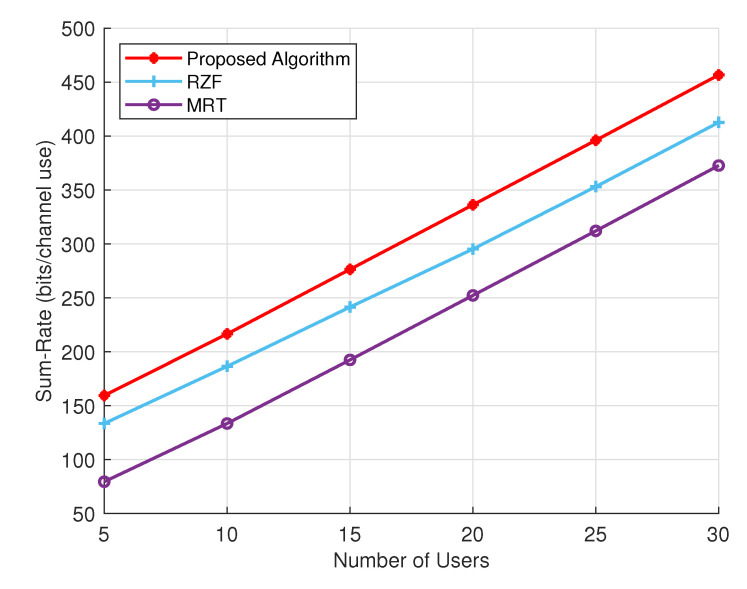
Sum rate of different precoding schemes with increasing number of users.

Figure 5 demonstrates a comparison of the sum rate achieved for different numbers of users, shedding light on the behavior of the proposed algorithm and other techniques as the user count increases. As the number of users grows, the sum rate attained by the proposed algorithm also rises. For instance, at five users, the sum rate is approximately 160 bits per channel use, escalating to around 460 bits per channel use at 30 users. On the other hand, the RZF algorithm exhibits a similar trend of increasing sum rate with the number of users, though at a slower pace compared to the proposed algorithm. Specifically, when the number of users is 5, the sum rate hovers around 130 bits per channel use, climbing to approximately 410 bits per channel use at 30 users. While RZF outperforms MRT, it falls short of the proposed algorithm, particularly with a larger user base. In contrast, the MRT algorithm registers the lowest sum rate among the three algorithms. Although the sum rate does increase with the number of users, the rate of increase is more gradual in comparison to the other two methods. Such as, at five users, the sum rate stands at roughly 80 bits per channel use, eventually reaching around 370 bits per channel use at 30 users. The proposed algorithm outperforms the other two algorithms, especially when the number of users is large.

**Figure 6:**
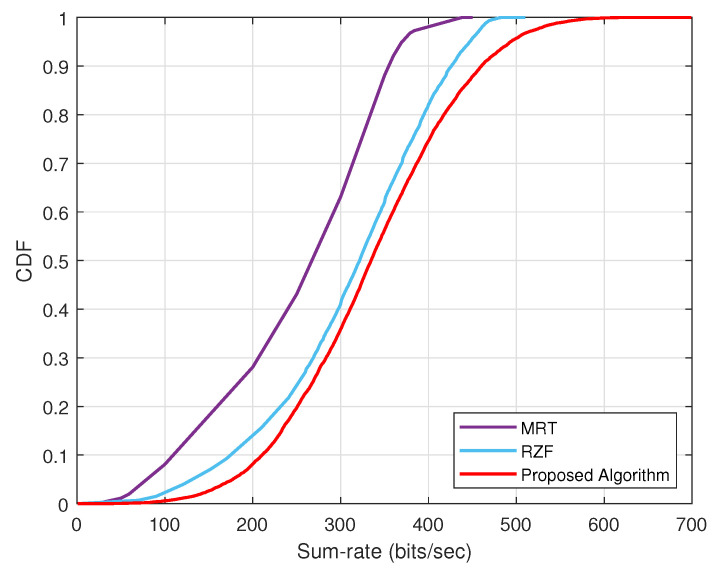
CDF of different techniqes with sum rate performance.

We conduct an analysis of the cumulative distribution function (CDF) for the sum rate for the MRT, RZF, and our proposed algorithm, as illustrated in Figure 6. The results show that at a probability of 1, the sum rate of MRT reaches 400, RZF hits 500, and the proposed algorithm obtains up to 700 bits/s. It is evident that MRT has the lowest probability of achieving a higher sum rate, while RZF outperforms MRT in terms of sum rate. Whereas, the proposed algorithm consistently demonstrates a superior CDF and has the highest probability of achieving a high sum rate, showcasing its better performance compared to the other algorithms.

Energy efficiency (EE) is a critical performance metric and is defined as the ratio of the sum rate to the total power consumption, including both the transmitted power and the power consumed by the system’s hardware and signal processing components. It is expressed as:

(15) $$EE=\frac{R}{P}.$$

**Figure 7:**
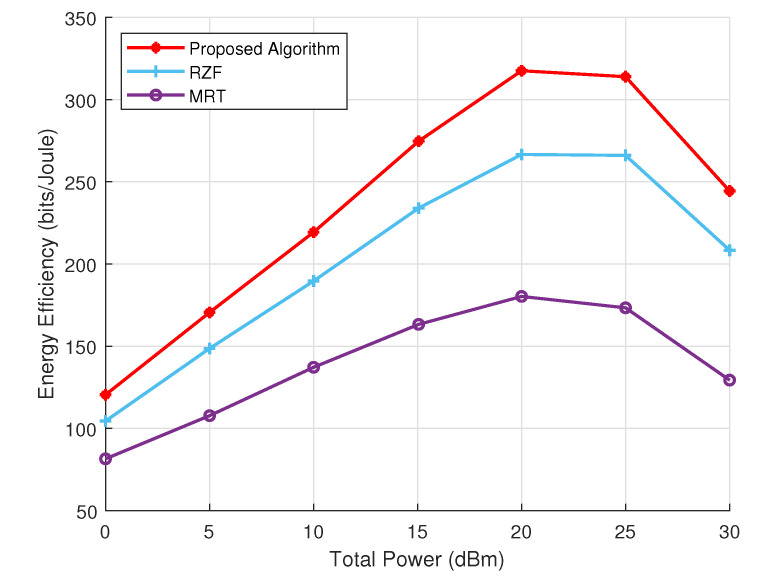
Energy Efficiency performance comparison.

Figure 7 presents a comparative analysis of the energy efficiency of the proposed algorithm, RZF, and MRT, highlighting the performance differences at various levels of total power. The proposed algorithm reveals notable energy efficiency across the entire power spectrum, achieving a peak rate of 317.55 bits per Joule. This performance surpasses the maximum efficiency of RZF (266.64 bits/Joule) and MRT (180.28 bits/Joule). Notably, all three methods exhibit a non-linear relationship between total power and energy efficiency, indicated by an initial rise, a distinct peak, and subsequent decline at higher power levels. The consistent superiority of the proposed algorithm, particularly its ability to maintain high efficiency even at high power levels, emphasizes its potential for substantial energy savings in the NM-MIMO-OWC system.

**Figure 8:**
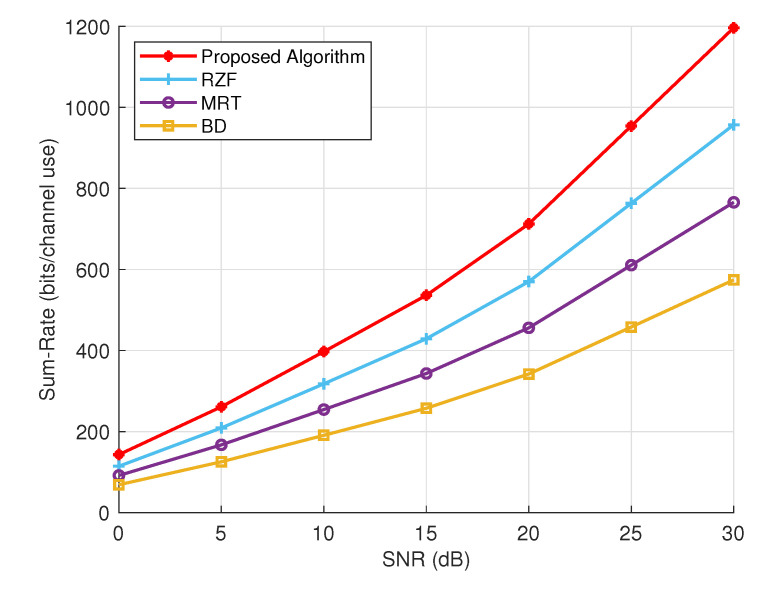
Comparative analysis of sum rate performance under expanded network settings and modified simulation parameters.

Conclusively, Figure 8 provides a comparative analysis of sum rate performance for various precoding techniques under expanded network settings and modified simulation parameters, which are listed in Table 2. The results indicate that the proposed algorithm consistently outperforms RZF, MRT and block diagonalization (BD) schemes, demonstrating the highest sum rates across all SNR levels, even with an increased number of transmit antennas. For instance, at a fixed SNR = 30 dBs, the proposed algorithm achieves a sum rate of 1196.48 bits/channel use, which is significantly higher than RZF (957.18), MRT (765.75), and BD (574.31). RZF shows the second-best performance, outperforming MRT and BD in all SNR values. At 30 dB, RZF achieves a sum rate of 957.18 bits/channel use, demonstrating its ability to effectively deal with inter-user interference while maintaining high spectral efficiency. MRT falls behind RZF but still provides better performance than BD, particularly at higher SNR values. At 30 dB, MRT reaches a sum rate of 765.75 bits/channel use. BD shows the lowest sum rates among the compared methods. At 30 dB, BD achieves a sum rate of 574.31 bits/channel use, indicating that while it effectively manages interference, it may not fully exploit the available SNR as efficiently as the other techniques.

## 6. Conclusions

We developed a system model for network massive MIMO OWC and employed precoding matrices to calculate the achievable rate. To maximize the sum rate while adhering to power constraints, we formulated an optimization problem and defined the objective function using the Lagrangian multiplier. By applying the KKT conditions, we solved the optimization problem and obtained an optimal precoder. Leveraging this optimality, we proposed a low-complexity iterative precoder design algorithm based on the bisection technique. We conducted a thorough analysis to assess the performance of the proposed technique in NM-MIMO-OWC systems with varying parameters. Our findings demonstrated that the proposed precoder design consistently achieves a higher sum rate under large numbers of optical antennas and users, outperforming other precoders. We also presented the convergence behavior of the proposed algorithm. Finally, through the validation of numerical results, it is evident that the proposed precoder design exhibits a performance advantage over other approaches. This highlighted the capability of the proposed optimization-based precoder design in maximizing the achievable sum rate while satisfying the power constraints.

## Figures and Tables

**Table 1:** Simulation Parameters.

Description	Value
Number of Base Stations	3
Number of Transmitters	128
Number of User Terminals	10
Number of Receivers	2
Consumed Power in dBm	30
Signal to Noise Ratio in dBs	20

**Table 2:** Expanded network settings and modified simulation parameters.

Description	Value
Number of Base Stations	6
Number of Transmitters	256
Number of User Terminals	100
Number of Receivers	6
Signal to Noise Ratio in dBs	20

## Data Availability

Data are contained within the article.

## Appendix A. Proof of Theorem 1

We calculate the Lagrangian as:

(A1) $$\begin{array}{cc} \; & L(P_{j,m},\lambda )=\\ & \sum_{i=1}^{U}\frac{1}{2}\{\log\det\left(\sum_{\ell }\sum_{k}H_{i,\ell }P_{k,\ell }P_{k,\ell }^{T}H_{i,\ell }^{T}+\sigma_{n}^{2}I\right)\\ & -\frac{1}{2}\log\det\left(\sum_{\ell }\sum_{k\neq i}H_{i,\ell }P_{k,\ell }P_{k,\ell }^{T}H_{i,\ell }^{T}+\sigma_{n}^{2}I\right)\}\\ & +\sum_{\ell }\lambda_{\ell }\left(\sum_{i=1}^{U}\mathrm{Tr}\left(P_{i,\ell }P_{i,\ell }^{T}\right)-P\right). \end{array}$$

From (A1) we find the partial derivative of L,

(A2) $$\begin{array}{cc} \; & \frac{\partial L}{\partial P_{j,m}}=\sum_{i}H_{i,m}^{T}{\left(\sum_{\ell }\sum_{k}H_{i,\ell }P_{k,\ell }P_{k,\ell }^{T}H_{i,\ell }^{T}+\sigma_{n}^{2}I\right)}^{-1}H_{i,m}P_{j,m}\\ & -\sum_{i\neq j}H_{i,m}^{T}{\left(\sum_{\ell }\sum_{k\neq i}H_{i,\ell }P_{k,\ell }P_{k,\ell }^{T}H_{i,\ell }^{T}+\sigma_{n}^{2}I\right)}^{-1}H_{i,m}P_{j,m}+\lambda_{\ell }P_{j,m}. \end{array}$$

The optimal precoder must meet the following conditions:

(A3) $$\frac{\partial L}{\partial P_{j,m}}=0,$$

(A4) $$\lambda_{\ell }\left(\sum_{i=1}^{U}\mathrm{Tr}\left(P_{i,\ell }P_{i,\ell }^{T}\right)-P\right)=0,$$

(A5) $$\lambda_{\ell }\geq 0,$$

(A6) $$\sum_{i=1}^{U}\mathrm{Tr}\left(P_{i,\ell }P_{i,\ell }^{T}\right)\leq P.$$

According to (A3) we have,

(A7) $$\begin{array}{c} \sum_{i}A_{i,m}P_{j,m}-\sum_{i\neq j}B_{i,m}P_{j,m}-\lambda_{\ell }P_{j,m}=0, \end{array}$$

where, $A_{i,m}$ and $B_{i,m}$ are given by:

(A8) $$A_{i,m}=H_{i,m}^{T}{\left(\sum_{\ell }\sum_{k}H_{i,\ell }P_{k,\ell }P_{k,\ell }^{T}H_{i,\ell }^{T}+\sigma_{n}^{2}I\right)}^{-1}H_{i,m},$$

(A9) $$B_{i,m}=H_{i,m}^{T}{\left(\sum_{\ell }\sum_{k\neq i}H_{i,\ell }P_{k,\ell }P_{k,\ell }^{T}H_{i,\ell }^{T}+\sigma_{n}^{2}I\right)}^{-1}H_{i,m}.$$

By rearranging (A7), the value of $P_{j,m}$ can be derived as:

(A10) $$\begin{array}{c} P_{j,m}={\left(\lambda_{\ell }I+\sum_{i}B_{i,m}-\sum_{i}A_{i,m}\right)}^{-1}B_{j,m}P_{j,m}. \end{array}$$

Hence, (A10) completes the proof.
